# Silver and Antimicrobial Polymer Nanocomplexes to Enhance Biocidal Effects

**DOI:** 10.3390/ijms25021256

**Published:** 2024-01-19

**Authors:** Diana Pereira, Susana Ferreira, Gloria Belén Ramírez-Rodríguez, Nuno Alves, Ângela Sousa, Joana F. A. Valente

**Affiliations:** 1 CICS-UBI-Health Sciences Research Centre, University of Beira Interior, Avenida Infante D. Henrique, 6200-506 Covilhã, Portugal; diana.carvalho.pereira1@gmail.com (D.P.); susana.ferreira@fcsaude.ubi.pt (S.F.); 2 Department of Inorganic Chemistry (BioNanoMetals Group), Facultad de Ciencias, Universidad de Granada, Avenida Fuente Nueva, s/n, 18071 Granada, Spain; gloria@ugr.es; 3 CDRSP-PL-Centre for Rapid and Sustainable Product Development, Polytechnic of Leiria, Marinha Grande, 2430-028 Leiria, Portugal; joana.valente@ipleiria.pt

**Keywords:** silver nanoparticles, chitosan, poly-L-lysine, epsilon-poly-L-lysine, dopamine, antimicrobial effect, bacteria, fungi

## Abstract

Antimicrobial resistance has become a major problem over the years and threatens to remain in the future, at least until a solution is found. Silver nanoparticles (Ag-NPs) and antimicrobial polymers (APs) are known for their antimicrobial properties and can be considered an alternative approach to fighting resistant microorganisms. Hence, the main goal of this research is to shed some light on the antimicrobial properties of Ag-NPs and APs (chitosan (CH), poly-L-lysine (PLL), ε-poly-L-lysine (ε-PLL), and dopamine (DA)) when used alone and complexed to explore the potential enhancement of the antimicrobial effect of the combination Ag-NPs + Aps. The resultant nanocomplexes were chemically and morphologically characterized by UV-visible spectra, zeta potential, transmission electron microscopy, and Fourier-transform infrared spectroscopy. Moreover, the Ag-NPs, APs, and Ag-NPs + APs nanocomplexes were tested against Gram-positive *Staphylococcus aureus* (*S. aureus*) and the Gram-negative *Escherichia coli* (*E. coli*) bacteria, as well as the fungi *Candida albicans* (*C. albicans*). Overall, the antimicrobial results showed potentiation of the activity of the nanocomplexes with a focus on *C. albicans*. For the biofilm eradication ability, Ag-NPs and Ag-NPs + DA were able to significantly remove *S. aureus* preformed biofilm, and Ag-NPs + CH were able to significantly destroy *C. albicans* biofilm, with both performing better than Ag-NPs alone. Overall, we have proven the successful conjugation of Ag-NPs and APs, with some of these formulations showing potential to be further investigated for the treatment of microbial infections.

## 1. Introduction

Pathogenic microorganisms are a major health safety issue due to their innate capacity for acquiring drug resistance [1,2]. These microorganisms develop resistance by enduring concentrations of antimicrobials that were once able to kill or inactivate them. Concerning this, antimicrobial resistance (AMR) has been recognized as a leading public health threat at the global scale by the World Health Organization [3,4,5]. It is estimated that about 4.95 million deaths were associated with bacterial AMR in 2019, including 1.27 million deaths attributable to bacterial AMR [4]. Primary causes of the emergence of these AMR infections can be attributed to the misuse and overuse of antibiotics in the human, animal, and environmental sectors [4,6,7]. In addition to the intrinsic or acquired mechanisms of resistance to antibiotics, biofilms present themselves as a microbial strategy that contributes to their ability to overcome antimicrobial activity [8]. Biofilms are an association of microorganisms encapsulated in a community by an extracellular matrix [8,9,10] and are strongly associated with healthcare persistent infections [9,10]. This kind of infection is acquired while patients receive healthcare treatments and is thought to be responsible for killing one in seventeen infected patients [11]. Despite the vast efforts made to develop new treatments to fight pathogens, there is still a need to overcome the difficulties related to their resistance [2].

Many strategies have been explored to fight AMR pathogens, such as the use of metals, like silver (Ag), which has been investigated for hundreds of years for its outstanding biocidal properties [12,13,14]. With the rise of nanotechnology, silver nanoparticles (Ag-NPs) have emerged and are applied to medical devices, water treatments, and domestic uses, among others, for protection against several pathogens [15]. The mechanism by which Ag-NPs exert antimicrobial activity has not been well-established yet. However, it is thought that when in contact with bacteria and fungi, their small size helps to provide a large surface area to contact the microbial cell walls. Once the contact has been made, there is a discharge of Ag+ ions that causes disturbance of the membrane and penetrates the cell. When inside the cell, they will generate reactive oxygen species (ROS) and free radicals that lead to damage to the DNA, stopping its replication and causing apoptosis [16].

Although Ag-NPs possess outstanding antimicrobial properties, they can be potentiated when combined with other antimicrobial compounds, such as antimicrobial polymers (APs) [17]. APs possess a wide range of antimicrobial mechanisms of action, which is an advantage compared to standard antimicrobial drugs that are normally directed toward a more specific target. In this way, it becomes harder for microorganisms to develop resistance against APs compared with conventional antimicrobial drugs [18,19]. Like Ag-NPs, many APs act on interference with the microbial cell walls and destabilize the pathogen membranes [19].

Among the different APs, chitosan (CH) is a well-known biodegradable and biocompatible AP that exhibits low toxicity [20,21]. Particularly, low molecular weight CH presents strong antimicrobial activity, with great solubility at the physiological pH, which is highly explored in the biomedical and pharmaceutical fields [22,23,24]. The formation of electrostatic interactions with the microbial cell walls, leading to damage or alteration of the membrane surface, has been proposed as one of the mechanisms responsible for the CH antimicrobial effect [25]. Another proposed antimicrobial mechanism of CH is the chelation of Ca^2+^ and Mg^2+^ ions, which weakens the cytoplasmic membrane [25]. Lastly, it is also suggested that CH can penetrate the cell nucleus, bind itself to nucleic acids, and inhibit the replication and transcription of DNA [25].

Poly-L-lysine (PLL) is a biocompatible AP that is used in the environmental and food industry fields [26,27]. PLL can perform electrostatic interactions with negatively charged molecules, such as DNA, and protect them against enzymatic degradation. This polymer can also enhance cellular internalization and has more applicability in drug delivery fields than in biocidal applications [28,29]. PLL can be classified into α-poly-L-lysine and ε-poly-L-lysine (ε-PLL). The latter compound has been widely researched in recent years owing to the improved antimicrobial activity and enhanced membrane selectivity compared to α-PLL due to the presence of entropically restricted cationic α-amine groups and isopeptide bonds [27,30]. α-PLL contains a long chain of 50 L-lysine residues with linkages by standard peptide bonds and presents inferior biocidal activity compared to ε-PLL. In turn, ε-PLL is a hydrophilic cationic linear polymer, composed of 25 to 35 identical L-lysine residues with peptide bonds between the α-carboxyl and ε-amino group of L-lysine-adjacent residues. Therefore, some authors stated that L-lysine polymerization via an isopeptide bond is a requirement for the biological activity of the polymer [27,30,31,32]. Moreover, ε-PLL presents thermo-stability, biodegradability, water solubility, biocompatibility, edibility, and nontoxicity, and it has also native antimicrobial activity and is suited for application in food protection, agriculture, and biomedical fields [27,33]. Regarding its antimicrobial action, ε-PLL can be electrostatically absorbed onto the microbial cell surface, affecting its integrity and permeability. Once inside the cell, it promotes ROS production that will induce oxidative stress responses and damage the DNA, affecting cell viability and leading to its death [33,34,35]. Furthermore, ε-PLL possesses cell selectivity, which has been attributed to the ability to recognize bulk charges present on the membrane surface of microorganisms that comprise anionic phospholipids, and it is, therefore, able to target prokaryotic over eucaryotic organisms [30,36].

Another interesting molecule presenting antimicrobial properties is dopamine hydrochloride (DA), which is an endogenous biomolecule in the catecholamine and phenylamine families used to correct hemodynamic disorders associated with shock episodes [37,38]. DA is also a biomaterial that can be deposited onto various material surfaces, improving their cohesiveness and functionalization, since it contains catechol and amino molecules that can self-polymerize and form polydopamine (polyDA) [39,40]. PolyDA presents unique features, such as strong adhesive properties on all kinds of surfaces, rich chemical functionalities, and biocompatibility suited for biological applications [40,41,42,43]. Recently, polyDA coatings have been widely researched due to their antimicrobial properties. During DA oxidation/functionalization in polyDA, there is a generation of ROS that have microbicidal properties, improving antimicrobial efficiency [42,43,44].

Due to the remarkable antimicrobial properties of Ag-NPs and APs, there was the possibility that together, these materials can exert an enhanced antimicrobial effect [18]. Therefore, this work aimed to explore the development of an organic (APs)/inorganic (Ag-NPs) nanoconjugate obtained by electrostatic interactions. The evaluation of the antimicrobial effect of these nanoconjugates was compared with the isolated compounds to search for evidence of enhanced antimicrobial behavior.

## 2. Results and Discussion

### 2.1. Characterization of Ag-NP + AP Coatings Using UV-Vis Spectra Measurements

**Figure 1:**
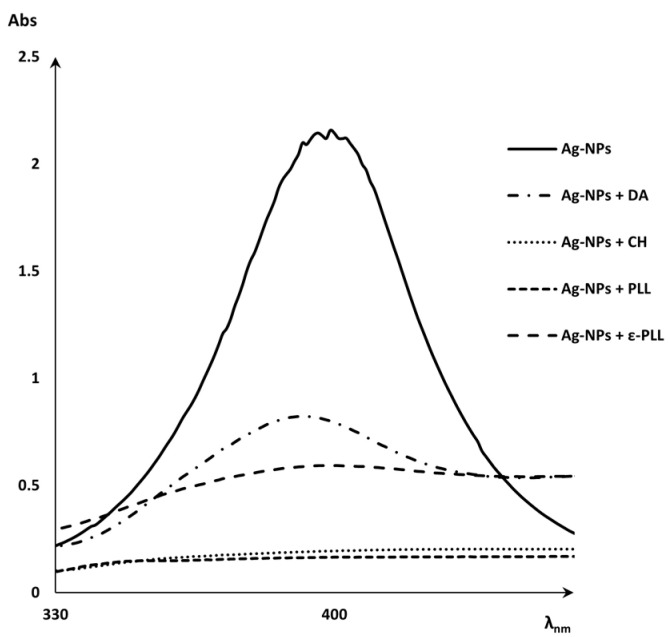
UV-vis spectra of Ag-NPs peak and Ag-NPs + APs (CH, DA, PLL, and ε-PLL).

To first evaluate the Ag-NPs + APs nanoconjugate formulation, UV-vis spectra of the Ag-NPs and Ag-NPs + APs were obtained before centrifugation (Figure 1). This analysis was performed to search for evidence of the coating of Ag-NPs by the polymers through a reduction in the silver peak. From a practical perspective, this reduction represents the absence of free Ag-NPs on the supernatant due to the binding with the polymers [45,46,47].

**Figure 2:**
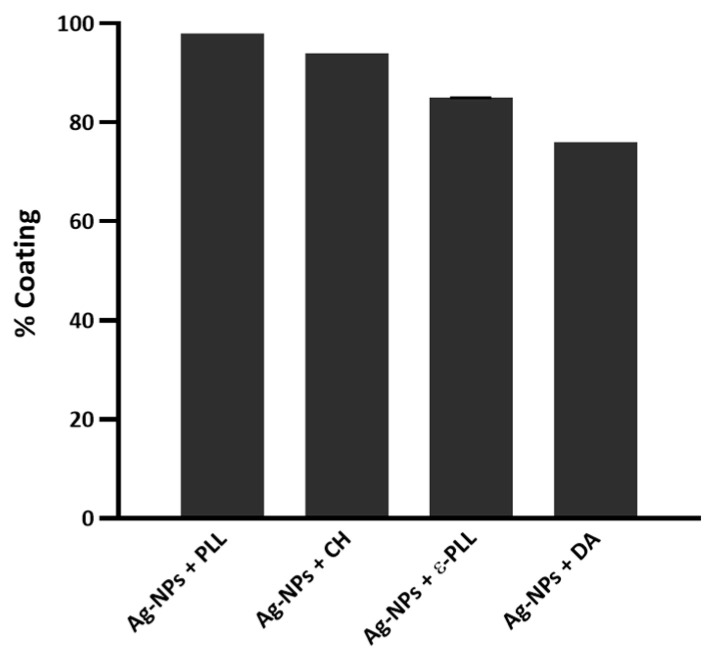
The yield of Ag-NPs coated with the different APs.

Then, the coating yield of Ag-NPs by APs was calculated (Figure 2), and it was observed that (i) after 2 h of incubation, the Ag-NP coating effect with PLL was about 98%; (ii) the CH polymer also presented a good performance, which coated approximately 94% of Ag-NPs after 4 h of incubation; (iii) for the Ag-NPs + ε-PLL, the coating effect reached 85% after 2 h of incubation; and lastly, (iv) DA coated about 76% of Ag-NPs.

It is thought that the coating efficiency of the Ag-NPs by PLL could be due to the formation of multiple polar bonds, and so PLL has largely been used to promote cell adhesion. The results showed that PLL can produce a stronger coating effect on the Ag-NPs surface than ε-PLL; however, the specific reason for this behavior remains unknown [48,49,50]. Nonetheless, both APs presented a rather satisfying coating ability after only 2 h of incubation. CH is known as an AP that is a strong chelating agent, and it has been widely used as a stabilizer to prevent the agglomeration of Ag-NPs [51,52]. In this work, the coating ability of CH was tested with 0 min and 30 min and 2 and 4 h of incubation, reaching an optimal coating performance after 4 h of incubation. Concerning the DA, it was observed that once incubated for some time, Ag-NP + DA formulations started to show a brownish color, indicating possible oxidization. Some authors refer to this occurrence as not desirable for clinical applications [53] and, therefore, we decided to eliminate the incubation period in this case. Overall, these coating results show that all polymers successfully coated the surface of Ag-NPs.

### 2.2. Morphological and Chemical Analysis of the Ag-NP + AP Nanocomplexes

**Figure 3:**
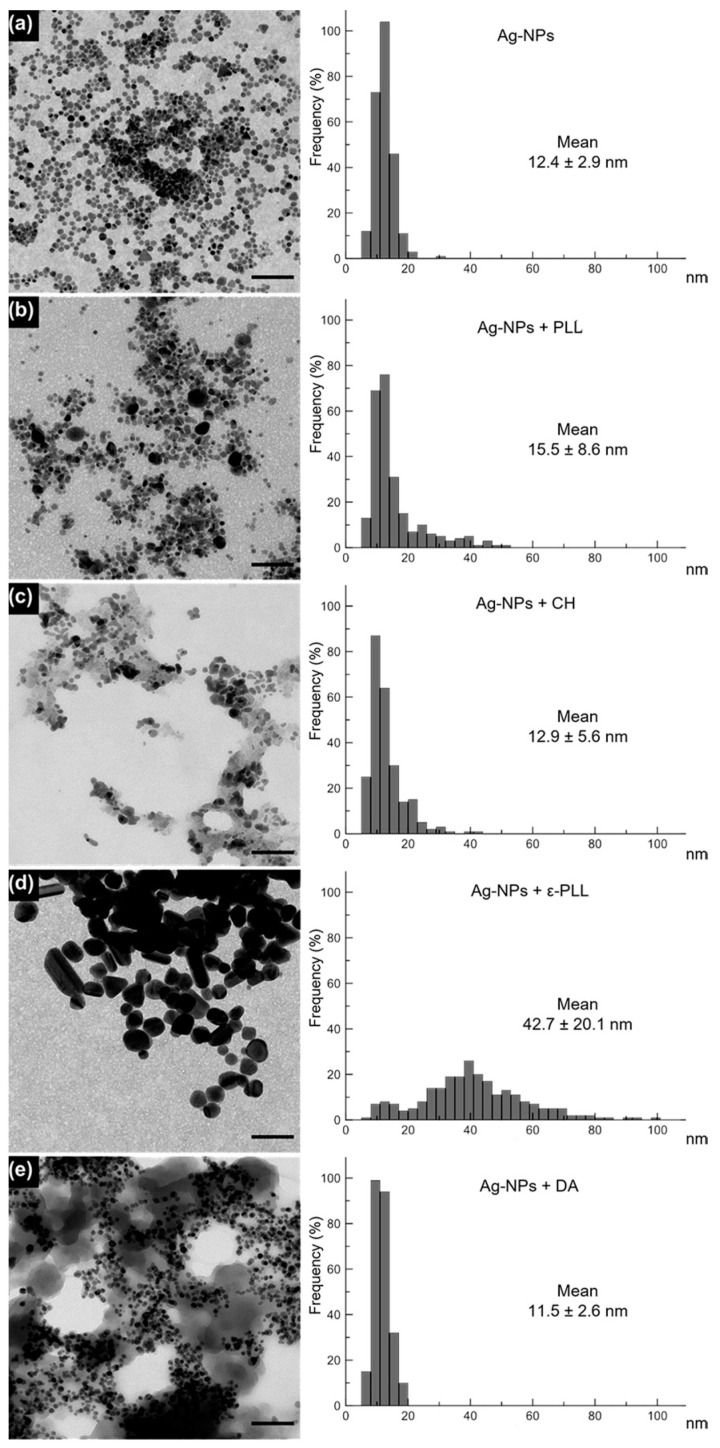
TEM images of Ag-NPs (**a**) and Ag-NPs coated with PLL (**b**), CH (**c**), ε-PLL (**d**), and DA (**e**) and a graphical representation of the diameter frequency and mean diameter by measuring 250 NPs for each sample. Scale bar = 100 nm. Histograms display the nanoparticle diameter distribution (nm) and mean size for each sample (n = 250 particles).

Polymer-coated Ag-NPs’ average size, as well as their morphology, were assessed through TEM analysis. Ag-NPs presented an average diameter size of 12.4 ± 2.9 nm, and after coating with PLL, CH, and DA, the average sizes remained similar and were obtained at 15.5 ± 8.6, 12.9 ± 5.6, and 11.5 ± 2.6 nm, respectively. Through the analysis in Figure 3, an enclosing shadow is observed, which suggests a polymeric coating. Additionally, all the formulations present a consistent and compact structure with spherical shapes, which indicates that they were able to maintain their shape and native size after the linkage with the polymer [54,55,56,57]. However, for ε-PLL, changes in shape and average diameter were observed (there was an increase to 42.7 ± 20.1 nm after the coating process). This effect could be due to the strong polycationic effect of ε-PLL, leading this polymer to interact with the Ag-NPs surface in several distinct positions, which could produce a ticker coating and consequent a size enlargement and shape deformity that could eventually hinder the nanocomplex performance in the following antimicrobial assays once the antimicrobial effect relies on the ability of ε-PLL to adhere to the bacterial membrane through the polycationic nature of the poly-peptide [27,58,59].

**Figure 4:**
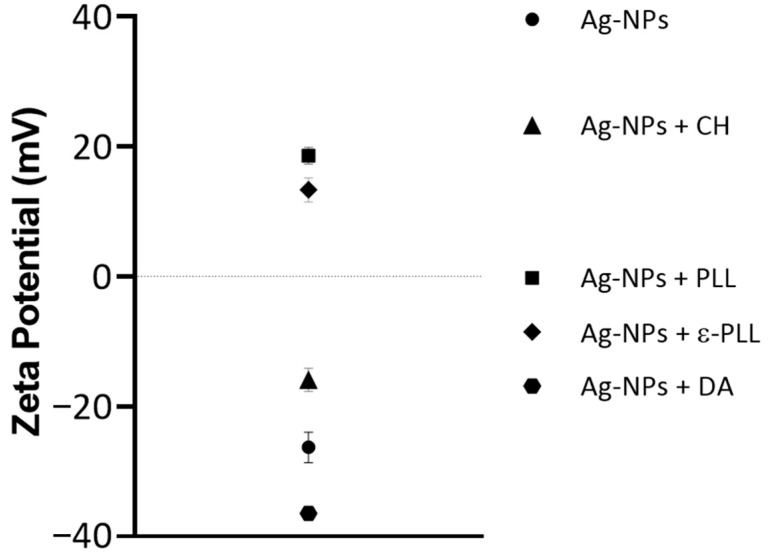
Average zeta potential values for Ag-NP and Ag-NP + AP formulations.

To further confirm the interaction between the polymer and Ag-NPs, the zeta potential of each formulation was measured (Figure 4). Ag-NPs initially have a negative zeta potential of −26.3 ± 4.053 mV (as expected due to the citrate coating). Still, their charge increases to 18.6 ± 2.170 and 13.3 ± 3.216 mV after PLL and ε-PLL coatings, respectively, which was also expected since these polymers are positively charged molecules [56,60]. With the CH coating, the charge also increases to −15.9 ± 3.079 mV; however, it remains slightly negative, which can be because NaOH was used in the formulation of Ag-NPs + CH. The pH was altered from acidic to alkaline, and the amino groups on CH might have lost their positive charge, with the coating becoming negatively charged [61,62]. Ag-NPs + DA present a zeta potential of −36.4 ± 1.097 mV since the DA molecule presents several phenolic and catechol functional groups that lead to a higher negative charge on the surface of NPs [63,64]. The changes in the zeta potential of the uncoated Ag-NPs to the Ag-NP + AP formulations are, therefore, further evidence of the Ag-NP surface modification and complexation with the polymers.

**Figure 5:**
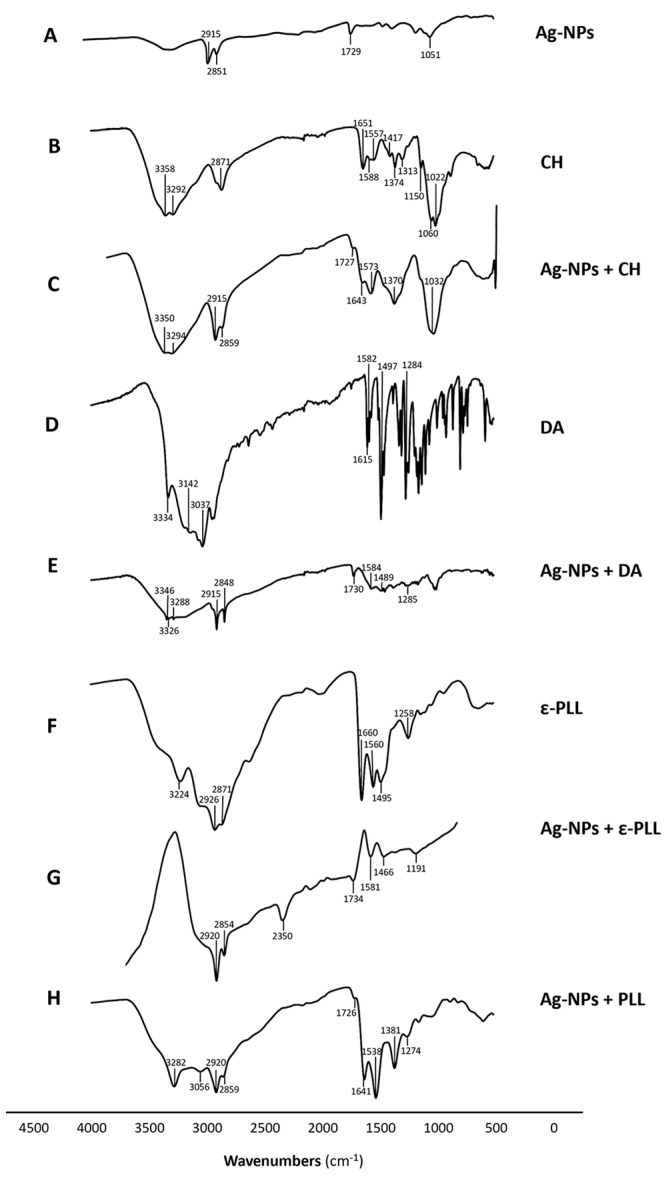
FTIR spectra of Ag-NP (**A**), CH (**B**), Ag-NP + CH (**C**), DA (**D**), Ag-NP + DA (**E**), ε-PLL (**F**), Ag-NP + ε-PLL (**G**), and Ag-NP + PLL (**H**) compounds and the signalized relevant peaks.

Moreover, an FTIR analysis of the NPs was performed to show the interaction between Ag-NPs and the polymers. In the obtained spectrum of Ag-NPs + CH (Figure 5C), it is possible to observe peaks in regions of 3350–3294 cm^−1^ corresponding to the stretching of N-H and O-H, as well as intramolecular hydrogen bonds. Peaks at 2859 cm^−1^ that correspond to C-H asymmetric stretching, peaks at 1643 cm^−1^ that correspond to C=O the stretching of amide I, peaks at 1573 cm^−1^ that correspond to the N-H bending of the primary amine, peaks at 1370 cm^−1^ that correspond to CH_3_ symmetrical deformations, and peaks at 1032 cm^−1^ that correspond to C-O stretching are coincident with the peaks found in the CH spectrum (Figure 5B) [65]. Beyond that, the Ag-NPs + CH spectrum also presents two other peaks at 2915 and 1727 cm^−1^ that, according to Goudarzi et al. (2016) and Chekuri et al. (2015), correspond to C-H symmetric stretching and C=O stretching of either lactones, ketones, or carboxylic anhydrides and are present in the Ag-NPs spectrum (Figure 5A) [66,67].

The Ag-NPs + DA (Figure 5E) spectrum presents peaks in regions of 3346–3288 cm^−1^ that correspond to amide N-H stretching. Peaks at 1584, 1489, and 1285 cm^−1^ correspond to primary amine stretching mode, the C-H bending of aromatic groups, and C-N stretching of amide III, respectively; that is, there is a correspondence to the peaks found in the DA spectrum (Figure 5D) and polyDA spectrum [68,69,70]. In addition, the Ag-NPs + DA spectrum also presents peaks in the region of 2915–2848 cm^−1^ and 1730 cm^−1^, which correspond to the C-H stretching and C=O stretching of either lactones, ketones, or carboxylic anhydrides, respectively, which are present in the Ag-NPs spectrum (Figure 5A) [67].

Ag-NPs + PLL (Figure 5H) and Ag-NPs + ε-PLL (Figure 5G) spectra present peaks at 3282–3311, 2859–2847, 1641–1648, 1538–1580, and 1274–1260 cm^−1^ that correspond to secondary amine stretching vibrations, symmetric stretching vibration of CH_2_, C=O stretching and vibrational modes of amide, N-H bending and vibrational modes of amide II/C–H stretching vibrations, and C–N stretching vibration of primary aliphatic amines/C-N stretching of amide III/C=O bending vibrations, respectively. These peaks are also present in the ε-PLL (Figure 5F) spectrum at 3224, 2871, 1660, 1560, and 1258 cm^−1^ and were also found to be present in the PLL spectrum, according to the findings of Miao et al. (2017) and Le et al. (2021) [71,72,73]. There is another peak present in the Ag-NPs + PLL spectrum at 1381 cm^−1^, which according to Goudarzi et al. (2016), represents CH_3_ symmetrical deformations present in the PLL spectrum [67]. The ε-PLL and Ag-NPs + ε-PLL spectra present other peaks at 1495 and 1492 cm^−1^, respectively, which correspond to vibrational modes of amide II [72]. Ag-NPs + PLL and Ag-NPs + ε-PLL spectra present peaks at 2920 and 1726–1715 cm^−1^, which correspond to the C-H symmetric stretching and C=O stretching of either lactones, ketones, or carboxylic anhydrides, respectively, which are found in the Ag-NPs spectrum (Figure 5A) [66,67].

Considering these results, it is possible to understand that all Ag-NP + AP nanoconjugates presented peaks from Ag-NP spectra and the respective polymer, which is another indicator of the interaction between the compounds.

### 2.3. Antimicrobial Activity of Ag-NPs and the Developed Nanoconjugates

To assess the antimicrobial activity of Ag-NPs and polymers alone and combined, MIC and MLC assays were performed against one representative member of Gram-positive and Gram-negative bacteria, as well as yeasts (Table 1). The initial concentrations of the polymers were adjusted accordingly to the coating percentage of Ag-NPs with the polymers and corresponded to 40 μg(Ag-NPs)/mL. For Ag-NPs alone, the MIC/MLC values were established as 20 µg/mL for *E. coli* and 40/>40 µg/mL for *S. aureus,* similar to the findings of Ansari et al. (2014), where they found the MIC of Ag-NPs with a 5–10 nm size against *E. coli* to be around 11.25–45 µg/mL and an MBC of 45 µg/mL. Mathew et al. (2018) found the MIC of Ag-NPs with a 4–15 nm size against *S. aureus* to be around 62.5 µg/mL, and the MBC was around 500 µg/mL [74,75]. No antifungal activity was detected for Ag-NPs against *C. albicans* in the tested concentration range, which is consistent with the observations from researchers showing that C. *albicans* tend to be more tolerant of Ag-NPs than other *Candida* species [76,77,78,79]. For instance, Khatoon et al. (2015) performed a study where they found that Ag-NPs showed a MIC of 30 μg/mL against *C. glabrata* and *C. tropicalis*, whilst for *C. albicans* (ATCC 90028), the MIC value doubled to 60 μg/mL [76]. Later, Khatoon et al. (2019) synthesized Ag-NPs through a different method but still found *C. albicans* to be the most tolerant species among the ones tested from the genus, needing a concentration of 120 μg/mL of Ag-NPs to reduce the growth by 90% compared to the 80 μg/mL and 90 μg/mL needed for *C. glabrata* and *C. tropicalis*, respectively [79]. These authors stated that the main difference between the effects on *C. albicans* compared to other species lies in the thickness and structural organization of the outer layer of the cell wall [76,79]. Aligned with what was previously stated, Mahnaz Masoumizadeh and co-workers (2022) also evaluated the effect of AgNPs against different *Candida* species and observed MIC values of 62.50, 31.25, and 15.62 mg/mL against *Candida albicans*, *Candida dubliniensis*, and *Candida guilliermondii*, respectively [77]. Additionally, on a broader scale, Carvalho Bernardo et al. (2021) compared the effects of Ag-NPs on *C. albicans* and various bacterial strains, such as *Actinomyces naeslundii*, *Fusobacterium nucleatum* subsp. *Nucleatum*, *S. aureus*, *Staphylococcus epidermidis*, *Streptococcus mutans*, *Streptococcus oralis*, and *Veillonella dispar*, and found that the bacterial strains were more susceptible to their Ag-NPs [78]. Moreover, an explanation for this phenomenon can be that *C. albicans* displays the ability to transition among different morphological forms, such as yeast, pseudohyphae, and hyphae, which is in agreement with what is stated above [80,81]. This morphological flexibility, also known as morphological plasticity, enables the fungus to adjust to diverse environments, enhancing its capacity to establish persistent infections and contribute to its resistance compared to other *Candida* species [80,81].

The CH polymer did not show activity for the tested bacteria or yeast in the concentration range evaluated, which is in line with the findings of Davoodbasha et al. (2018), who found MIC values for *E. coli* and *S. aureus* between concentrations of 80 and 160 µg/mL and high MIC values for *C. albicans* [82]. However, when CH was combined with Ag-NPs, the MIC values decreased, in comparison with Ag-NPs, to 10 µg/mL for *E. coli* and 20 µg/mL for *S. aureus*, which were lower than Ag-NPs and CH alone, which can be an indicator of the enhanced antimicrobial effect of the nanoconjugates. The observed improvement may potentially be associated with the fact that CH mucoadhesive properties helped the nanoconjugate attach itself to the bacterial cell wall, allowing a more effective penetration of silver ions into the cells. Furthermore, it is known that the CH polymer can interact with Ag-NPs due to the presence of amino groups and stabilize them through electrostatic interactions. These NPs can interact with the bacterial membranes, and the low molecular weight of CH has a more pronounced effect on Gram-negative bacteria [83,84]. Regarding the use of Ag-NPs combined with CH, the MIC value was 10 µg/mL for *C. albicans*, a concentration that was demonstrated to be fungicidal and substantially lower than Ag-NPs and CH alone. This result highlights the enhanced antimicrobial effect of these nanoconjugates.

The PLL polymer presented MIC values of 40 µg/mL for *E. coli* and >40 µg/mL for *S. aureus* and *C. albicans*. To our knowledge, there is not much information in the literature about the antibacterial activity of the PLL, but so far, our results demonstrate that the polymer does not have a relevant antimicrobial activity on its own. When the PLL was combined with Ag-NPs, there was no considerable enhanced effect of Ag-NPs + PLL; in turn, for *C. albicans*, the MIC and MLC were 20 µg/mL and 40 µg/mL, respectively. These results show that contrary to the antibacterial results, the antifungal effect of Ag-NPs + PLL was greater compared with each compound alone. On the other hand, ε-PLL is known for presenting strong antimicrobial activity [27], and it presents a much better effect when acting alone than in conjugation with Ag-NPs for bacteria and yeast. The main reason for this behavior could be that the amino side chain group, which is the source of the electrostatic interaction between the polymer and the microbial membrane, becomes less available due to the complexation with the Ag-NPs [27,59]. The effect of the Ag-NPs + ε-PLL on *C. albicans* (a MIC value of 20 µg/mL) contrasts with no activity against bacteria. This may be related to the different composition among yeast and bacterial cell walls that is possibly associated with a different affinity of the polymer to bacterial and yeast surface components. Nonetheless, more studies are required to further explore this hypothesis.

For DA, the MIC and MLC values were 40 µg/mL and 10 µg/mL for *E. coli* and *S. aureus*, respectively, and 40 µg/mL and >40 µg/mL for *C. albicans*. The results for the antibacterial effects of DA agree with what was found in the literature by Sushomasri et al. (2010), where a lower amount of DA was needed to inhibit the *S. aureus* strains compared with the DA needed to inhibit *E. coli* strains [85]. Moreover, for the Ag-NPs + DA combination, the MIC values were reduced for *S. aureus* and *C. albicans*, with MLC values of 40 µg/mL for *S. aureus* and 10 µg/mL for *C. albicans* compared with the non-conjugated Ag-NPs. Furthermore, the values for Ag-NPs + DA were lower than those obtained for DA, suggesting an enhanced antimicrobial behavior when the nanocomplexes are applied. According to the literature, arachidonoyl dopamine, which is a member of the N-acyl dopamine family produced by the conjugation of arachidonic acid and DA hydrochloride, was reported to interact with active site residues of a sterol 14-alpha demethylase enzyme [86,87]. The sterol 14-alpha demethylase enzyme plays a major role in the biosynthesis of ergosterol, which is important for the growth of fungi and essential for the synthesis of fungal cell membranes. Therefore, it is considered a molecular target for antifungal experiments, especially for *C. albicans* [88,89]. Even though azoles (important antifungal drugs) also target the 14-alpha demethylase enzyme, in this situation, DA can assist in interacting with the fungal cell wall. However, in this case, because they are combined with Ag-NPs, their antifungal action can be enhanced, leading to a stronger combined antifungal effect [87,90]. This could help to explain why our Ag-NPs + DA nanoconjugate had an enhanced effect on fungi.

Overall, differences in the antibacterial effect of our nanoconjugates were observed between Gram-positive *S. aureus* and Gram-negative *E. coli*, probably due to their morphological differences [2,91]. All the nanoconjugates, when active, maintained the bactericidal activity reported for Ag-NPs.

Regarding, the analysis of the antifungal experiments, it can be observed that all the nanoconjugates presented fungicidal effects. Moreover, all the combinations showed an improved effect compared with the Ag-NPs, which was more evident for Ag-NP + CH and Ag-NP + DA nanoconjugates.

#### Evaluation of the Biofilm Eradication Ability

Bacterial and fungal biofilms pose a serious threat to human health, as about 90% of bacteria grow in the form of biofilms [92,93,94]. These communities are known for forming an extracellular matrix that allows them to attach to different surfaces, whilst protecting them from adverse conditions and antimicrobials [92,93,94]. Their structure is difficult for antimicrobial compounds or other treatments to exert their effect successfully and, therefore, it is of utmost importance to find new strategies to fight them [92,93,94].

For the analysis of the effect of the selected nanoconjugates on preformed biofilms, we selected the strains for which the nanocomplexes presented an MIC reduction of at least four-fold of the one determined for Ag-NPs alone for the following experiments. Therefore, the Ag-NPs + CH activity was evaluated on the preformed biofilms of *S. aureus*, and Ag-NPs + CH and Ag-NPs + DA were evaluated on *C. albicans.*

**Figure 6:**
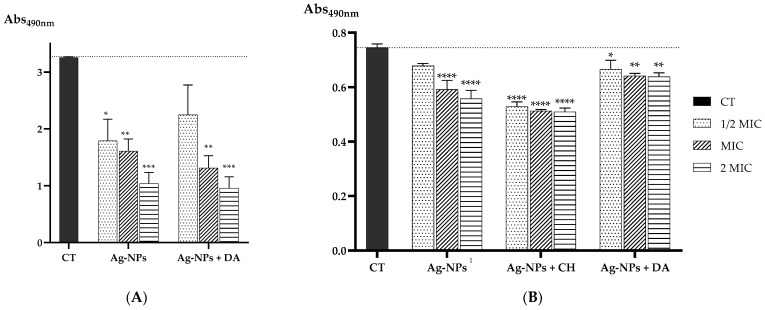
(**A**) Absorbance values at 490 nm of the biological activity of *S. aureus* biofilm after incubation with different concentrations of Ag-NP and Ag-NP + CH nanocomplexes; (**B**) the biological activity of *C. albicans* biofilm after incubation with different concentrations of Ag-NP, Ag-NP + CH, and Ag-NP + DA nanocomplexes. Data are presented as the mean, and the standard error of the means and the statistical significance were measured using one-way ANOVA software in GraphPad Prism version 8.4.3, obtaining * *p* < 0.05, ** *p* < 0.01, *** *p* < 0.001, and **** *p* < 0.0001 compared to the control (CT). ^1^ indicates that as the MIC was determined as >40 µg/mL, the used concentrations were calculated considering the values of 20 µg/mL for 1/2× MIC, 40 µg/mL for MIC, and 80 µg/mL for 2× MIC.

For the *S. aureus* strain (Figure 6A), Ag-NPs and Ag-NPs + DA presented a significant eradication of the biofilm; however, it should be noted that the concentrations of Ag-NPs + DA needed for a similar effect were about four-fold lower than the ones used for Ag-NPs. These findings are in line with some reports in the literature. For instance, in 2021, Xu and co-workers tested a nanocomposite based on polydopamine plus antimicrobial peptides and Ag-NPs against the same *S. aureus* strain, ATCC 25923. They found that their formulation was able to significantly destroy the formed biofilm compared to the control and were stronger than Ag-NPs alone [94]. In addition, Zhang and colleagues tested polydopamine-modified nanoparticles loaded with gentamicin in a different strain of *S. aureus* (CCUG10778), showing a significant biofilm viability decrease upon treatment, which can indicate that DA plays an important role in antibacterial effects [94,95].

Similarly, for the *C. albicans* strain (Figure 6B), a better effect was obtained for the nanoconjugates considering the use of 20, 40, and 80 µg/mL for Ag-NPs, 5, 10, and 20 µg/mL for Ag-NPs + CH, or even 2.5, 5, and 10 µg/mL for Ag-NPs + DA. Lower concentrations of nanoconjugates allowed a similar profile in the reduction in the preformed biofilm in comparison with Ag-NPs. From these results, we can gather that the Ag-NP + CH formulation seems to be the best in reducing the viability of the biofilm.

## 3. Materials and Methods

### 3.1. Ag-NPs Coating with APs

Silver dispersion 10 nm (0.02 mg/mL, 107.87 MW), Chitosan Low Molecular Weight (50,000–190,000 MW), and Poly-L-Lysine Hydrobromide (30,000–70,000 MW) were purchased from Sigma Aldrich, St. Louis, MO, USA; Poly-ε-L-lysine hydrochloride (2600–3800 MW) was purchased in Biosynth Carbosynth, Bratislava, Slovakia; and Dopamine Hydrochloride (99%, 189.64 MW) was acquired from Thermo Fisher Scientific, Dreieich, Germany.

For Ag-NPs + CH, 1 mg/mL of CH solution was prepared in 10 mL of PBS, and HCl 5 M was added to achieve pH 4.5. Then, the solution was left stirring for 24 h at room temperature and was filtered at 0.22 μm and stored at room temperature. Afterward, 50 μL of CH was mixed with 250 μL of silver dispersion and 43 μL of NaOH in the vortex, incubated at room temperature for 4 h [45]. Ag-NPs + PLL were prepared by performing a 1 mg/mL PLL solution in ultrapure water and stored at −20 °C. Afterward, 250 μL of silver dispersion was mixed under stirring with 50 μL of PLL and incubated at room temperature for 2 h [48,96]. For Ag-NPs + ε-PLL, a solution of 10 mg/mL ε-PLL in ultrapure water was prepared. Then, 50 μL of ε-PLL was used for complexation with 250 μL of silver dispersion, stirred in the vortex, and incubated at room temperature for 2 h [48,96]. In the case of Ag-NPs + DA, a solution of 2 mg/mL of DA was prepared in Tris-HCl 10 mM pH 8.5. A volume of 100 μL was mixed with 250 μL of silver dispersion in the vortex [97,98]. All the formulations were centrifuged twice at 10,000× *g* for 20 min and, followed by a dilution of the deposit in ultrapure water for use in the characterization processes.

### 3.2. Morphological and Chemical Characterization

UV–visible (UV-vis) spectra were obtained through a scan reading of each of the different samples, Ag-NPs and Ag-NPs + APs, in a SPECTROstar Nano (BMG Labtech, Ortenberg, Germany) using a quartz cell with 10.0 mm of the optical path. To evaluate the decrease in the Ag-NPs peak when coated with each polymer, a graphical representation of the different spectra before centrifugation was obtained [45].

The coating yield of the Ag-NPs by the polymers was determined by measuring each sample of Ag-NPs + AP at 400 nm and determining it using a calibration curve of Ag-NP concentration in the range between 0.1 and 2 µg/mL versus its absorbance at 400 nm measured in a NanoPhotometer^TM^ (Implen, Munich, Germany) (y = 0.391x − 0.0041; r2 = 0.9996).

The morphology of the Ag-NPs + APs was recorded by transmission electron microscopy (TEM) images using a LIBRA 120 PLUS instrument (Carl Zeiss SMT, Oberkochen, Germany) from the Centre for Scientific Instrumentation of the University of Granada (CIC-UGR, Granada, Spain), operating at 120 kV. Ag-NPs and Ag-NPs + APs were ultrasonically dispersed in ultrapure water, and then a drop of the slurry was deposited on 200 mesh copper grids covered with thin amorphous carbon films. After 5 min of incubation, the grids were washed thrice with ultrapure water (20 μL drops) and left to air dry under ambient conditions. A total of 250 nanoparticles were measured for each condition with ImageJ2 software. This experiment was performed to assess if the Ag-NPs preserved their native size and if the coating process did not promote particle aggregation.

The chemical composition of Ag-NPs and polymer complexes was also investigated by Fourier-transform infrared spectroscopy (FTIR) analysis. All the samples were lyophilized overnight, and the spectra were acquired using a Nicolet iS10 FTIR spectrophotometer (Thermo Scientific, Waltham, MA, USA) with an average of 120 scans, a spectral width ranging from 4000 to 525 cm^−1^, and a spectral resolution of 32/4 cm^−1^.

The zeta potential of the Ag-NPs and Ag-NPs + APs was measured using a Zetasizer Nano ZS (Malvern Instruments, Malvern, UK), and Malvern Zetasizer software v 6.34 was used to obtain and analyze the data.

### 3.3. Antimicrobial Activity

Müller–Hinton agar (MHA) was from VWR Chemicals, Leuven, Belgium; Müller–Hinton broth (MHB) was from Liofilchem, Roseto degli Abruzzi, Italy; Sabouraud dextrose agar (SDA) was from Oxoid Ltd., Altrincham, UK; Roswell Park Memorial Institute 1640 (RPMI 1640) came from Gibco^®^, Paisley, UK; and Tryptic Soy Broth (TSB) and Yeast Extract–Peptone–Dextrose (YPD) liquid media were acquired from VWR, Leuven, Belgium. The antibiotics tetracycline (Tet) and amphotericin B (Amp B) were from Sigma Aldrich, St. Louis, MO, USA. The 3-(4,5-dimethylthazolk-2-yl)-2,5-diphenyl tetrazolium bromide (MTT) was obtained from Alfa Aesar (Waltham, MA, USA).

#### 3.3.1. Microorganisms and Culture Media

The bacterial species used were the Gram-positive *Staphylococcus aureus* (*S. aureus*) ATCC 25923 and the Gram-negative *Escherichia coli* (*E. coli*) ATCC 25922. For the fungi species, *Candida albicans* (*C. albicans*) ATCC 90028 was used. The *S. aureus* and *E. coli* species were grown in MHA overnight at 37 °C, and the assays were performed in MHB medium. For *C. albicans*, the media used for overnight growth at 37 °C were SDA, and RPMI 1640 medium supplemented with 3-(N-morpholino)propanesulfonic acid (MOPS) was used for assays.

#### 3.3.2. Determination of the Minimum Inhibitory Concentration (MIC) and Minimum Lethal Concentration (MLC)

The combined and isolated susceptibility of the bacteria and fungi to the Ag-NPs and polymers were evaluated by the microdilution method. The inoculum was prepared by suspension in NaCl 0.85% (*w*/*v*) by adjusting the turbidity to 0.5 McFarland and diluting with medium to achieve a final cell concentration of approximately 5 × 10^5^ CFU/mL for bacteria and 1–5 × 10^3^ CFU/mL for fungi [99,100]. Tetracycline and amphotericin B were used as the controls for these experiments.

The assays were performed with concentrations ranging between 40 and 0.3125 μg/mL for polymers, Ag-NPs and Ag-NPs + polymers, and 4 and 0.03125 μg/mL for both tetracycline and amphotericin B. The concentrations for the combination Ag-NPs + polymers considered the Ag-NPs present. The microplates were then incubated at 37 °C for 24 h for bacteria and 48 h for fungi. The MIC was defined as the lowest concentration leading to the absence of visible growth to the naked eye. The MLC was determined by subculturing 20 µL of culture to agar plates, in each well where visible growth was prevented, and incubating at 37 °C for 24 h. The MLC was defined as the lowest concentration of the compound leading to a ≥99.9% reduction in the initial inoculum. At least three independent determinations were performed, and the results were presented as modal values.

#### 3.3.3. Antibiofilm Studies

To access the Ag-NP, Ag-NP + CH, and Ag-NP + DA ability to eradicate *S. aureus* and *C. albicans* preformed biofilms, the strains were grown overnight at 37 °C in an MHA and SDA medium, respectively. The overnight cultures of *S. aureus* and *C. albicans* were prepared in TSB supplemented with 0.5% glucose or YPD medium, respectively, and incubated at 37 °C and 250 rpm. Afterward, 100 µL of the cultures of *S. aureus* and *C. albicans* strains with a concentration of 1.0 × 10^8^ CFU/mL and 1.0 × 10^6^ CFU/mL, respectively, were added to the 96-well plates in TSB supplemented with 0.5% glucose or RPMI 1640 supplemented with MOPS, respectively, and were incubated for 24 h at 37 °C. After this period, the wells were washed twice with PBS, and the Ag-NP, Ag-NP + CH, and Ag-NP + DA formulations were added in concentrations of 1/2× MIC, 1× MIC, and 2× MIC, or a medium was used as the control, and the plates were re-incubated at 37 °C for another 24 h. Afterward, the wells were washed twice with PBS, and 100 µL of 1 mg/mL of MTT diluted in the respective medium was added to each well and incubated in the dark at 37 °C for 4 h. Then, each well was washed twice with PBS and 150 µL of DMSO was added, incubated for 20 min, and 100 µL of each well was added to a new 96-well plate to measure the absorbance at 490 nm for *S. aureus* and *C. albicans* [101,102].

Four independent experiments were performed, and each condition was performed in quadruplicate. Then, the data were analyzed using a one-way ANOVA statistical test, where each sample was compared to the control, and the *p*-values are presented as follows: * *p* < 0.05, ** *p* < 0.01, *** *p* < 0.001, and **** *p* < 0.0001.

## 4. Conclusions

The present research aimed to combine the strong antimicrobial properties of Ag-NPs and APs through electrostatic interactions to form nanoconjugates and evaluate their antimicrobial behavior as nanoconjugates or isolated compounds to search for evidence of antimicrobial-enhanced behavior.

Initially, coating yield measurements were performed to guarantee that all the APs successfully coated the Ag-NP surface. From these results, it was observed that PLL and CH polymers presented a stronger coating ability of 98% and 94%, respectively. Morphological experiments showed that the average diameter size of the original Ag-NPs was maintained, and aggregation was only observed for Ag-NPs + ε-PLL. Additionally, the nanoconjugates were also chemically characterized by a measurement of zeta potential and FTIR experiments, indicating the success of the conjugation of all APs with the Ag-NPs.

Furthermore, antimicrobial assays were performed against both Gram-positive (*S. aureus*) and Gram-negative (*E. coli*) bacteria strains and yeast strains (*C. albicans*). All nanoconjugates presented lower MIC and MLC values than the individual APs or Ag-NPs, except for Ag-NPs + ε-PLL, which had a better effect when ε-PLL was used alone. However, we also evaluated the nanocomplex’s ability to destroy biofilm formation. These results showed that Ag-NPs + DA was able to destroy more efficiently biofilm than Ag-NPs alone for both *S. albicans* and *C. albicans* biofilms, with a more significant effect on *S. aureus*. The effect of Ag-NPs + CH on *C. albicans* was more significant than Ag-NPs + DA.

Regarding the obtained results, Ag-NPs + CH and Ag-NPs + DA formulations seem to have the most consistent and pronounced antimicrobial effects, but overall, conjugating APs with Ag-NPs leads to an enhanced antimicrobial effect compared with these compounds alone, making these conjugates good candidates for biocidal treatments.

In a world that faces the challenges of rising antimicrobial-resistant pathogens, there is an urgent need to find better alternatives for standard treatments. Concerning this, the present research aimed to combine the strong antimicrobial properties of Ag-NPs and APs through electrostatic interactions to form nanoconjugates and evaluate their antimicrobial behavior as nanoconjugates or isolated compounds to search for evidence of antimicrobial-enhanced behavior. Overall, we demonstrated that APs, like CH and DA, when combined with Ag-NPs, can produce two powerful formulations to fight pathogenic microorganisms and help remove their preformed biofilms. In the future, it would be interesting to test these systems against other types of microorganisms or even combine them with therapeutic compounds and use them as delivery systems for therapeutic approaches. As a future perspective, it would be of interest to study the effect of Ag-NPs + DA against azole-resistant *C. albicans* strains and further explore their mechanism of action.

## Figures and Tables

**Table 1:** The MIC and MLC of AP, Ag-NP, and Ag-NPs + AP.

Compound	MIC/MLC (µg/mL)		
	***E. coli***	***S. aureus***	***C. albicans***
Ag-NPs	20/20	40/>40	>40/ND *
Ag-NPs + CH	**10**/20 *	**20/40** *	**10/10** *
CH	>40/ND *	>40/ND *	>40/ND *
Ag-NPs + PLL	40/40	40/**40** *	**20/40** *
PLL	40/40	>40/ND	>40/ND *
Ag-NPs + ε-PLL	>40/ND *	>40/ND *	**20/40** *
ε-PLL	1.25/1.25	2.5/2.5	0.625/5
Ag-NPs + DA	20/20	**10/40** *	**5/10** *
DA	40/40	10/10	40/>40
Tet	1/>4	0.5 >4	-
Amp B	-	-	0.25/0.25

* ND—not determined; Ag-NPs +APs in (µg Ag-NP/mL). Changes in the MIC or MLC relative to the Ag-NPs are marked in bold.

## Data Availability

The raw data required to reproduce these findings cannot be shared at this time as the data also form part of an ongoing study.
